# Novel Single Nucleotide Polymorphism Markers for Low Dose Aspirin-Associated Small Bowel Bleeding

**DOI:** 10.1371/journal.pone.0084244

**Published:** 2013-12-18

**Authors:** Akiko Shiotani, Takahisa Murao, Yoshihiko Fujita, Yoshinori Fujimura, Takashi Sakakibara, Kazuto Nishio, Ken Haruma

**Affiliations:** 1 Division of Gastroenterology, Department of Internal Medicine, Kawasaki Medical School, Kurashiki City, Okayama, Japan; 2 Department of Genome Biology, Kinki University Faculty of Medicine, Sayama City, Osaka, Japan; 3 Department of Gastroenterology, Sakakibara Heart Institute of Okayama, Okayama City, Okayama, Japan; Mathematical Institute, Hungary

## Abstract

**Background:**

Aspirin-induced enteropathy is now increasingly being recognized although the pathogenesis of small intestinal damage induced by aspirin is not well understood and related risk factors have not been established.

**Aim:**

To investigate pharmacogenomic profile of low dose aspirin (LDA)-induced small bowel bleeding.

**Methods:**

Genome-wide analysis of single nucleotide polymorphisms (SNPs) was performed using the Affymetrix DMET™ Plus Premier Pack. Genotypes of candidate genes associated with small bowel bleeding were determined using TaqMan SNP Genotyping Assay kits and direct sequencing.

**Results:**

In the validation study in overall 37 patients with small bowel bleeding and 400 controls, 4 of 27 identified SNPs: CYP4F11 (rs1060463) GG (*p*=0.003), CYP2D6 (rs28360521) GG (*p*=0.02), CYP24A1 (rs4809957) T allele (*p*=0.04), and GSTP1 (rs1695) G allele (*p*=0.04) were significantly more frequent in the small bowel bleeding group compared to the controls. After adjustment for significant factors, CYP2D6 (rs28360521) GG (OR 4.11, 95% CI. 1.62 -10.4) was associated with small bowel bleeding.

**Conclusions:**

CYP4F11 and CYP2D6 SNPs may identify patients at increased risk for aspirin-induced small bowel bleeding.

## Introduction

Aspirin had long been regarded as safe beyond the duodenum because of its rapid absorption and lack of an enterohepatic recirculation [1]. However, in recent studies, small bowel injury and enteropathy associated with low dose aspirin (LDA) are increasingly being recognized [2-5]. In a recent Japanese retrospective cohort study of patients taking LDA, the incidence of overt GI bleeding and suspected of small bowel blood loss was 3.9% (upper GI 3.1%, colon 0.7%, and small bowel 0%) and 1.4%, respectively [6]. In another retrospective study of patients undergoing LDA therapy following percutaneous coronary intervention (PCI), the incidence of GI bleeding within 30 days after PCI was 4.3% including occult bleeding (1.9%), small bowl ulcer (0.5%), and overt not upper GI bleeding(0.5%) indicating that attention must be paid to small bowel and lower GI bleeding as well as upper GI bleeding [7].

LDA is now widely used for primary and secondary prevention of cardiovascular events [8-10]. The thienopyridine derivatives, ticlopidine or clopidogrel are also regarded as mandatory following PCI at least until the coronary stents are fully endothelialized which requires approximately 3 months for bare metal stents and up to 1 year for drug-eluting stents [11]. One concern regarding prolonged antiplatelet or anticoagulant (antithrombotic) therapy is the risk of gastrointestinal (GI) bleeding including bleeding from the small intestine [10-13]. However, the pathogenesis and risk factors for small bowel damage induced by aspirin are not also well understood making prevention difficult.

In this era of personalized medicine, the technology allows one to identify genetic risk factors in relation to side effects of medical therapy. To date, there are few studies of the association between genetic polymorphisms and the risks of aspirin-induced ulcer or its complications[14-17]. Individuals with two single nucleotide polymorphisms (SNPs) of cyclooxygenase-1 (COX-1), A-842G and C50T, exhibit increased sensitivity to aspirin and lower prostaglandin synthesis capacity but the polymorphism lacked statistical significance in relation to an association with bleeding peptic ulcer [18]. Our recent Japanese study suggested that the *SLCO1B1* 521TT genotype and the *SLCO1B1* *1b haplotype were significantly associated with the risk of peptic ulcer and ulcer bleeding in patients taking low dose enteric-coated aspirin [17]. However, data on the related factors with small intestinal or lower GI events among the patients taking LDA are lacking. Particularly, to our knowledge the association between genetic markers and risk of small bowel bleeding induced by NSAIDs including LDA are still lacking. Therefore, the aim of the present study was to identify the genetic markers related with small bowel bleeding among the patients taking LDA.

## Materials and Methods

Study subjects consisted of patients taking 100 mg of enteric coated aspirin (Bayer Health Care, Osaka, Japan) and suspected of bleeding from the small intestine and controls. All patients with at least a one year history of aspirin and or at least a 3 month history of anti-thrombotics use were entered. The study was conducted in accordance with the Declaration of Helsinki from 2007 January to 2012 April at the hospital of Kawasaki Medical School and Sakakibara Heart Institute of Okayama, Japan. The permission was granted by the Ethics Committees of both hospitals, and written informed consent was obtained from each patient.

### Subjects

Patients who had complaints of fresh GI bleeding or exacerbating anemia with positive fecal occult blood test had undergone abdominal ultrasonography, upper GI endoscopy, and total colonoscopy. If the patients had no identified source for GI bleeding, bleeding from the small intestine was suspected. All patients with suspected bleeding from the small intestine underwent video capsule endoscopy (VCE) within one month and the diagnosis of aspirin induced enteropathy was made by VCE findings such as multiple erosions and/ or ulcers. Outpatients taking 100 mg of enteric-coated aspirin who had no complaint of GI bleeding or exacerbating anemia and had no identified source for bleeding or peptic ulcer by upper GI endoscopy were included as controls. Patients were excluded if they had lesions identified as causing small bowel bleeding such as malignant, tumorous, inflammatory, or vascular lesions. Patients were also excluded if they had gastric cancer or other malignant lesions. 

Demographic data were collected at entry included age, gender, alcohol and smoking consumption, and drug treatments including doses and internal use periods. These data were collected by interview using structured questionnaires and from the patients’ clinical records. The most evaluated medicines were continued more than 3 years including aspirin, and the all evaluated medicines were confirmed to be unchanged from others within 2 months. The medicines which had been prescribed just before endoscopy or VCE were not evaluated.

### Genotyping

Genomic DNA was extracted from 200 μL of peripheral blood using QIAamp DNA Blood Mini kits (QIAGEN GmbH, Hilden, Germany). 

#### Genome-wide analysis of SNPs

Seventeen patients taking enteric coated LDA and suspected bleeding from small intestine and 18 patients taking aspirin without bleeding and peptic ulcer who were matched with age, sex, medicine taken and diseases were enrolled. Genome**-**wide analysis of SNPs was performed using Affymetrix DMET**™** Plus Premier Pack. The DMET™ Plus GeneChip array (Affymetrix Inc, Santa Clara, CA) contains 1931 SNPs and five Copy and number Variants (CNVs) distributed on 225 drug metabolizing enzymes and transporters genes. Amplified and non-amplified DNA samples were combined for the annealing and amplification steps, in which molecular inversion probes (MIP) technology was exploited to genotype all the genomic sites of interest in a single reaction. DNA samples were subsequently purified, fragmented, labeled and hybridized to the array to be scanned with the Gene Chip Scanner 3000 (Affymetrix Inc, Santa Clara, CA).

#### Quality control

Before proceeding to the analysis, we performed quality control checks on the data. First, we tested the concordance between the genetic and reported sex to check for errors in labeling the samples. Second, all subjects showing a genotype call rate <95% were removed. Third, SNPs mapping on the regions of interest (i.e. containing the drug metabolism genes, about 6000 SNPs) were removed if there Hardy-Weinberg p-value was less than 0.00001.

#### Database submission of microarray data

The microarray data were deposited in the Gene Expression Omnibus (GEO) database: http://www.ncbi.nlm.nih.gov/geo/.  The GEO accession number for the platform is GSE52155.

#### Validation of the candidate SNPs

Genotypes of candidate genes associated with small bowel bleeding were determined by using TaqMan SNP Genotyping Assay kits (Life Technologies, Carlsbad, CA) by following the manufacturer’s instructions and were confirmed by direct sequencing. 

### Video capsule endoscopy(VCE)

Subjects fasted for 12 h before swallowing the video capsule and data were collected for 8 h-16h after ingestion. Water and lunch of choice were permitted 2 h and 4 h after capsule ingestion, respectively. Two well-trained physicians who remained blinded to the patients’ groups separately reviewed each of the procedures for intestinal injury and identified all suspected lesions by recording as thumb-nail photographs using RAPID® Access 5 or 6.5 software (Given Diagnostic Imaging System, Given Imaging, Tokyo). Each procedure was separately reviewed by recording as thumb-nail photographs. Adjudication was performed by at least four investigators simultaneously reviewing the thumb-nail photographs and by knowledge of the results of other diagnostic imaging such as double-balloon enteroscopy, ultrasonography, computed tomography, etc. The patients were asked to repeat VCE, if procedure was incomplete because of a large quantity of residue or blood and persistence of the capsule in the upper GI.

### Analysis

Values were expressed as the mean + standard deviation (SD). The differences of age and body mass index were analyzed by unpaired t test, and Mantel-Haenszel statistics were used to assess the differences in the other demographic and clinical characteristics. The odds ratio (OR) and 95% confidence interval (CI) were obtained by Mantel-Haenszel statistics and multiple logistic regression analysis to identify the risk or preventive factors after adjustment for the other significant factors determined by univariate analysis. Differences in the genotype frequencies between the two groups and Hardy-Weinberg equilibrium of allele frequencies at individual loci by comparing the observed and expected genotype frequencies were assessed using the chi-square test or the Fisher’s exact probability test. A two-sided *p* value of less than 0.05 was considered statistically significant. All statistical computations were performed using SPSS (version 11.0 for Windows, SPSS Inc, Chicago, IL).

## Results

### Patients’ characteristics

The genome**-**wide analysis group consists of 17 of the 37 patients with bleeding and 18 of the 400 controls of the study, and a total of 437 Japanese patients (287 men and 150 women; 42-91years old; average age 71 years) were enrolled. The study groups consisted of 37 patients with suspected bleeding from small intestine (the bleeding group) and 400 controls. Demographic and clinical characteristics are shown in Table 1. Age, Sex, drinking, or body mass index, complication of diabetes mellitus or renal failure was not significantly different between the small bowel bleeding group and controls (Table 1). The prevalence of active smokers was significantly higher (35.1% vs. 11%, p<0.001) in the bleeding group compared to controls. The percentage of the patients with ischemic heart disease treated with aspirin was significantly lower (56.8% vs. 73.5%, p=0.03) and those with non-cardiac vascular diseases including cerebrovascular diseases (24.3% vs. 6.8%, p<0.001) were significantly higher in the bleeding group compared to controls. The percentages of the patients taking warfarin (43.2% vs. 26%, p=0.03) and NSAIDs (16.2% vs. 3.0%, p<0.001) in the bleeding group were significantly higher than those in controls, but the other medicines including proton pomp inhibitor (PPI) were not associated with small bowel bleeding (Table 1). 

**Table 1:** Demographic and clinical characteristics of the patients.

Variable	Controls	Bleeding	*P*
	n=400 (%)	n=37 (%)	
Mean age yr (SD)	70.9(8.4)	72.1(10.3)	0.43
Over 80 yr of age (%)	67(15.7)	8(23.5)	0.23
Sex Male (%)	263(65.8)	24(73)	0.47
Active alcohol drinking (%)	118(29.5)	9(24.3)	0.83
Active smoking (%)	44(11)	13(35.1)	<0.001
Body mass index (SD)	23.5(3.1)	23.0 (4.0)	0.40
Ischemic heart diseases (%)	294(73.5)	21(56.8)	0.03
Other cardiac diseases (%)	88(22)	6(16.2)	0.67
Cerebrovascular diseases (%)	27(6.8)	9(24.3)	<0.001
Other vascular disease (%)	14(3.5)	6(16.2)	<0.001
Diabetes mellitus (%)	115(28.8)	11(29.7)	0.84
Chronic renal failure (%)	6 (1.5)	2(5.4)	0.13
Warfarin	104(26)	16(43.2)	0.03
Thienopyridine	111(27.8)	13(35.1)	0.34
PPI	145(36.3)	13(35.1)	0.90
H2-RA	133(33.3)	9(24.3)	0.27
Ca-blocker	158(39.5)	11(29.7)	0.25
ARB or ACE inhibitor	231(57.8)	20(54.1)	0.66
Statin	209(52.3)	15(40.5)	0.17
Nitrite	113(28.3)	13(35.1)	0.37
NSAID	12(3.0)	6(16.2)	<0.001

*p* Values; age and body mass index by unpaired t test and others by Mantel-Haenszel Chi square analyses. *p* Values by Mantel-Haenszel Chi square analyses; Thienopyridine, ticlopidine 200 or 300 mg/day or clopidogrel 50 or 75 mg/day; PPI, proton pomp inhibitor, omeprazole 10 mg/day, lansoplazole 15mg/day; H2-RA, H2-receptor antagonist, famotidine 10 or 20mg/day; Ca-blocker, nifedipidine 20 or 40mg/day, amlodipine 2.5mg or 5mg/day; ACE inhibitor, angiotensin-converting enzyme inihibitor, imidapril 5mg/day; ARB, angiotensin type 1 receptor blocker, candesartan 4 or 8 mg/day, telmisartan 40mg/day, olmesartan 20mg/day; Statin (HMG-Co A reductase inhibitor), pravastatin 10mg/day, atorvastatin 10mg/day, Rosuvastatin 5mg/day; Nitirite, carvedilol 20 mg/day, metoprolol 40mg/day; NSAID, Non-steroidal anti-inflammatory drugs internal use only.

### Candidate SNPs discriminated by DMET

Among the 1,936 SNPs included in the DMET system, we obtained the genotyping data of 1,771 SNPs with 100 % call rate; 1,215 SNPs were identical in all patients tested. As a result, we used genotyping data of the remaining 556 SNPs for statistical analysis. The SNPs detected to be significantly associated with small bowel bleeding using DMET included 27 SNPs of 23 genes and are listed in Table 2.

**Table 2:** Lists of discriminating polymorphisms associated with enteropathy using DMET.

Common Name	Chromosome	dbSNPRS ID	Alleles	*p* value
*CYP2A7_c.821A>G(H274R)*	19	rs4079366	A // G	0.000919
CYP2B6*27_15708T>C(M198T)	19	rs36079186	C // T	0.003325
*XDH_c.3030C>T(F1010F)*	2	rs1884725	C // T	0.003889
CYP2B6*6_15631G>T(Q172H)	19	rs3745274	T // G	0.004337
CYP2B6*4_18053A>G(K262R)	19	rs2279343	A // G	0.006724
SLC10A2_c.*315G>T	13	rs279941	T // G	0.009141
*SLC10A2_c.511G>T(A171S)*	13	rs188096	G // T	0.009141
*GSTA2_c.335G>C(S112T)*	6	rs2180314	G // C	0.010715
*GSTA5_c.-31+2057C>T*	6	rs4715354	C // T	0.010715
NAT2*7_c.857G>A(G286E)	8	rs1799931	A // G	0.018691
*SLC22A11_c.1058+487G>A*	11	rs1783811	A // G	0.021517
*GSTA1_c.-5120T>G*	6	rs4715332	T // G	0.025858
*CYP2D6_-2178G>A*	22	rs28360521	G // A	0.027304
CYP2A6*9_-48T>G(Promoter)	19	rs28399433	T // G	0.027991
*SLCO3A1_c.1513-5136A>G*	15	rs2283458	A // G	0.031028
*CYP4F11_4927T>C(I106I)*	19	rs3765070	C // T	0.034569
*CYP4F11_20043G>A(D446N)*	19	rs1060463	G // A	0.034569
ABCG1_c.*1981G>A	21	rs1541290	A // G	0.034569
UGT2B15_c.*168C>T(3'UTR)	4	rs3100	T // C	0.036341
CYP2F1*4_112T>C(S38P)	19	rs58285195	C // T	0.036704
*CYP11B2_3496A>G(A291A)*	8	rs4536	G // A	0.036819
*CHST2_2082C>T*	3	rs6664	C // T	0.0378
GSTP1*B_c.313A>G(I105V)	11	rs1695	A // G	0.0387
*CYP2A7_c.931C>T(R311C)*	19	rs3869579	T // C	0.039033
*ALB_c.-400G>A*	4	rs3756067	A // G	0.040307
*CYP24A1_18948C>T*	20	rs4809957	T // C	0.04416
*SLC22A8_c.723T>A(T241T)*	11	rs2276299	T // A	0.048146

### Validation study

The candidate SNPs were successfully evaluated in the total of 437 patients, and 5SNPs of 4 genes were significantly associated with small bowel bleeding (Tables 3, 4). The CYP4F11 20043G>A (D446N) and 4927T>C(I106I) alleles were in almost complete linkage disequilibrium. The frequencies of the CYP4F11 20043GG (70.3% vs. 43.5%, p=0.003), CYP2D6 -2178GG (45.9% vs. 25.2%, p=0.02), CYP24A1 18948 T allele (86.5% vs. 68%, p=0.04), and GSTP1*Bc.313 G allele (37.9% vs. 23.3%, p=0.04) were significantly higher in the bleeding subjects than in controls (Table 4).

**Table 3:** Allele and genotype frequencies of the candidate genes.

Genotype		Allele frequencies	Controls	Bleeding	*p*
*CYP4F11*	GG	G=0.66	174(43.5%)	26 (70.3%)	0.007
*20043G>A(D446N)*	GA	A=0.34	178(44.5%)	9 (24.3%)	
*rs1060463*	AA	p ^a^ =0.82	48 (12%)	2 (5.4%)	
*CYP2D*	GG	G=0.52	101(25.2%)	17(45.9%)	0.05
*-2178G>A*	GA	A=0.48	205(51.3%)	15(40.5%)	
*rs28360521*	AA	p ^a^ =0.85	94(23.5%)	5 (13.5%)	
*CYP24A1*	TT	C=0.43	65 (16.2%)	7(20%)	0.09
*18948C>T*	CT	T=0.57	207(51.8%)	25(67.6%)	
*rs4809957*	CC	p ^a^ =0.61	128 (32.8%)	5(13.5%)	
GSTP1*B	AA	A=0.86	307 (76.7%)	23(62.1%)	0.07
*c.313A>G(I105V)*	AG	G=0.14	86 (21.5%)	12 (32.4%)	
*rs1695*	GG	p ^a^ =0.90	7 (1.8%)	2 (5.4%)	

*p* Values by using the chi-square test; a, Hardy-Weinberg equilibrium (HWE) of allele frequencies at individual loci was assessed by comparing the observed and expected genotype frequencies.

**Table 4:** Association of the candidate polymorphisms with the risk of aspirin induced enteropathy.

Genotype	Controls	Bleeding	OR (95%C.I.)	*p*
*CYP4F11 20043*	174 vs 226	26 vs 11	3.08	0.003
*GG vs GA & AA*	43.5% vs 56.5%	70.3% vs 29.7%	(1.48-6.39)	
*CYP2D6 -2178*	101 vs 299	17 vs 20	2.35	0.02
*GG vs GA & AA*	25.2% vs 74.8%	45.9% vs 54.0%	(1.15 - 4.80)	
*CYP24A1 18948*	272 vs 128	32 vs 5	2.82	0.04
*CT &TT vs CC*	68% vs 32%	86.5% vs 13.5%	(1.07-7.42)	
GSTP1*Bc.313	93 vs 307	14 vs 23	2.08	0.04
*AG & GG vs AA*	23.3% vs 76.7%	37.9% vs 62.1%	(1.03-4.23)	

OR, odds ratio. *p* Values, odds ratio (OR) and 95% confidence interval (CI) were obtained by Mantel-Haenszel statistics.

### Factors associated with small bowel bleeding

Smoking (adjusted OR 8.46, 95% CI 3.09-23.2), non-cardiac vascular diseases (6.59, 2.48-17.5), co-treatment of warfarin (3.59, 1.41-9.14), and GG homo-genotypes of CYP2D6 -2178 rs28360521 (4.11, 1.62-10.4) were significantly associated with small bowel bleeding in multivariate analysis after adjustment of the significant factors in univariate analysis (Table 5). NSAIDs, GG homo-genotypes of CYP4F11 20043 rs1060463, and GSTP1*Bc.313 G allele rs1695 were not significantly associated with small bowel bleeding.

**Table 5:** Association between various relating factors and aspirin induced enteropathy.

	Unadjusted OR (95%CI)	Adjusted OR (95%CI)
Smoking	6.13 (2.80 - 13.4)***	8.46 (3.09 - 23.2) ***
Non-cardiac vascular disease	4.41(1.90- 10.2)**	6.59 (2.48- 17.5) **
Warfarin	2.15 (1.08 -4.27)*	3.59 (1.41 -9.14)**
NSAID	6.18 (2.20 - 17.4)**	1.66 (0.28 - 10.0)
*CYP4F11 20043 GG*	3.08 (1.48-6.39)**	2.09(0.80 - 5.44)
*CYP2D6 -2178 GG*	2.35 (1.15 - 4.80)*	4.11 (1.62 - 10.4)**
*CYP24A1 18948*	2.82 (1.07 - 7.42)*	3.40(0.90 - 13.0)
GSTP1*Bc.313	2.08 (1.03 - 4.23)*	1.46(0.56 - 3.80)

The unadjusted odds ratio (OR) and 95% confidence interval (CI) were obtained by Mantel-Haenszel statistics and the adjusted OR and 95%CI by multiple logistic regression analysis after adjustment for the other factors. *, *p*<0.05; **, *p*<0.01; ***, *p*<0.001.

## Discussion

Our study attempted to identify genetic risk factors associated with small bowel bleeding. Although 27 candidate SNPs of 23 genes were identified by DMET™ Plus GeneChip array, in our validation study, LDA associated small bowel bleeding occurred more often in the patients carrying the GG homo-genotypes of CYP4F11 (rs1060463) or CYP2D6 (rs28360521) , the patients carrying T allele of CYP24A1(rs4809957), or G allele of GSTP1 (rs1695). The role of the other SNPs must await additional studies with larger numbers of samples than available from our array study.

 Human CYP4F11 is one of the orphan cytochrome P450 with limited information regarding heterologous expression and functional characterization. It has been reported to be located in a cluster with the other five members of the Cytochrome P450 4F (CYP4F) subfamily on chromosome 19p13.1-2; its mRNA is expressed mainly in human liver [19]. Cytochrome P450 4F (CYP4F) enzymes are involved in cellular protection and metabolism of numerous small molecules, including drugs, toxins, and eicosanoids [20]. CYP4F11 appears to be involved in arachidonic acid and fatty-acid metabolism and catalyzes ω-hydroxylation of leukotriene B4, lipoxins A4 and B4, and hydroxyeicosatetraenoic acids (HETEs) (ie, metabolites with roles in many biological processes including platelet aggregation). Although CYP4F11 activities are much lower than those of CYP4F3 [21], CYP4F11 may have a role in platelet aggregation which would provide a theoretically basis for it being related to small bowel bleeding induced by LDA. However, its function is still not well characterized and there was no study investigating the biologic basis of CYP4F11 SNPs in this study. The SNP of CYP4F11 (rs1060463) was the most significantly associated with small bowel bleeding among the identified SNPs. However, in multivariate analysis it lost significance as well as taking NSAIDs which is well known risk factor for small bowel injury and bleeding. There may be some interactional association between NSAIDs use and the CYP4F11 SNP.

CYP2D6 plays a critical role in drug metabolism and synthesis of cholesterol, steroids and other lipids. This protein is localized to the endoplasmic reticulum and is known to metabolize as many as 20% of clinically prescribed drugs such as psychotropics, antihistamine, beta-blockers etc., but not aspirin or NSAIDs [22]. The CYP2D6 gene is polymorphic and almost 80 variant alleles have been reported. CYP2D6*3, CYP2D6*4, and CYP2D6*5 are the major alleles involved in the poor metabolizers (PM) that have little enzymatic activity, while CYP2D6*10 is a variant allele of the intermediate metabolizer (IM) phenotype. The frequency of PMs is less than 1% and IM phenotype is markedly dominant in Asia including the Japanese population. In the present study, the SNP associated with small bowel bleeding was CYP2D6 rs28360521, which is found -2182 5’-UTR of the *CYP2D6* gene. This SNP is in linkage disequilibrium with the entire gene (as well as 2 other more distant genes: *NDUFA6* and *NAGA*) [23,24]. The rs28360521 may be considered as potentially etiologic, although it is unknown how it might play a role in small bowel bleeding induced by LDA.

We first reported the possible association of CYP4F11 and CYP2D6 SNPs with LDA induced small bowel bleeding, and there is no report indicating the association of the SNPs with not only small bowel bleeding, but also upper GI bleeding. Moreover, we failed to confirm the significant association of the previously identified SNPs related with LDA induced peptic ulcer with small bowel bleeding (Data is not shown). The pathogenesis of small intestinal damage induced by NSAIDs including aspirin is not well understood. Based on the animal studies and in vitro studies, the same mechanisms in the stomach such as topical irritant properties of NSAIDS inducing direct mucosal toxicity, mitochondrial damage, breakdown of intercellular integrity has been assumed [25]. However, enterohepatic recirculation, which is prevented by ligation of bile ducts seems to be more important component of the mechanism underlying the pathogenesis of small intestinal damage than suppression of prostaglandin synthesis [26,27]. Moreover, enteric bacteria is thought to cause and exacerbate the intestinal damage elevating intestinal permeability by NSAIDs, activating neutrophils, generating induction of inducible nitric oxide synthesis [28-30]. The further studies how the SNPs are involved in LDA induced enteropathy or bleeding are required.

In the present study, active smoking, non-cardiac vascular diseases including cerebrovascular diseases warfarin, and NSAIDs were significantly associated with small bowel bleeding among the patients taking LDA. Drug combinations involving antiplatelets and anticoagulants are known to lead to a greatly increased risk of GI bleeding, and the prescribing of aspirin with warfarin was reported to increase risk of GI bleeding than that observed with each drug alone [31]. There is no report indicating association of small bowel bleeding with smoking. Moreover, there is no evidence indicating that the prevalence of GI bleeding was more frequent in aspirin users with non-cardiac vascular diseases compared to those with cardiac disease, and the risk of GI bleeding seems to be similar according to the previous studies although there is no data comparing the risk of small bowel bleeding [32]. The significant results of underlying disease treated by LDA possibly were caused by selection bias. Wallace JL et al [30] recently reported PPIs significantly exacerbated NSAIDs-induced intestinal ulceration and bleeding in the rat. However, in the present study there was no association between PPI use and small bowel bleeding. The limitation of our study is case control and the small number of patients with small bowel bleeding, and the large scale clinical cohort studies are absolutely required to investigate the possibility of this important issue. 

Although our data needs to be validated and extended in a larger cohort, this exploratory study suggests that CYP4F11 and CYP2D6 SNPs may identify patients at increased risk for aspirin-induced small bowel bleeding.
